# Instagram Use and Body Dissatisfaction: The Mediating Role of Upward Social Comparison with Peers and Influencers among Young Females

**DOI:** 10.3390/ijerph19031543

**Published:** 2022-01-29

**Authors:** Federica Pedalino, Anne-Linda Camerini

**Affiliations:** 1 Faculty of Communication, Culture and Society, USI Università della Svizzera italiana, 6900 Lugano, Switzerland; federica.pedalino@gmail.com; 2 Institute of Public Health, USI Università della Svizzera italiana, 6900 Lugano, Switzerland

**Keywords:** body dissatisfaction, Instagram, upward social comparison, influencers, females, structural equation modeling

## Abstract

Background: Instagram is one of the most popular social media platforms among young females. Idealized body images shared on the platform have been associated with lower levels of body satisfaction in this population, likely due to social comparison processes. In the present study, we tested a mediation model linking Instagram use (i.e., browsing through others’ profiles, commenting on others’ looks, posting one’s own photos or stories) to body dissatisfaction (i.e., body image discrepancy and lack of body appreciation), mediated by upward social comparison with close peers, distant peers, and social media influencers. Methods: We applied structural equation modeling to self-report cross-sectional data collected from 291 female adolescents and young women (M_age_ = 19.8, SD = 4.6; 94.8% Italian). Results: Our final model results show that browsing on Instagram was associated with lower levels of body appreciation, fully mediated by upward social comparison with social media influencers, not close or distant peers. Commenting on others’ looks and posting own content were not associated with body dissatisfaction. Being an adolescent female (compared to a young woman) and having a higher BMI were associated with worse body appreciation. Conclusions: Our findings highlight the need for public health interventions to raise awareness about the posting practices of social media influencers and to strengthen a positive body image among young females susceptible to social comparison processes.

## 1. Introduction

In September 2021, the Wall Street Journal published the Facebook Files, uncovering that the platform owner conducted their own research and found that Instagram is making body issues worse for one in three teenage girls, mainly because Instagram contributes to unhealthy social comparison among teens [1]. Instagram is one of the most popular social media platforms among the younger population, especially female adolescents and young women. As of October 2021, 3.7% of its users were females aged 13–17, whereas 13.1% were women between the ages of 18 and 24 years old [2]. The platform is mainly used for sharing photos, videos, and stories that people can edit by applying filters or other creative tools before they post them on their accounts [3]. On Instagram, users not only interact with peers, but also follow celebrities and social media influencers [4]. Since Instagram content is characterized by a positivity bias, where users generally present an idealized image of themselves [5,6], researchers have suggested even before the publication of the Facebook Files that Instagram may be more detrimental to women’s body image than any other social media platform, such as Facebook, which presents more varied content [7]. Prior studies on the link between Instagram use and women’s body dissatisfaction mainly focused on social comparison behaviors with peers [8] and specific features such as the role of “selfies” [9] or the number of “likes” [10]. Recent research also considered Instagram influencers [11], focusing on adult women.

The current study was designed to investigate the relationship between Instagram use and body dissatisfaction among female adolescents and young women. In particular, the study considered (1) different types of Instagram use (i.e., browsing, commenting, posting), (2) the mechanism through upward social comparison, and (3) the role of close and distant peers as well as social media influencers as comparison targets.

### 1.1. Instagram Use and Body Dissatisfaction

Instagram includes a plethora of (seemingly) authentic pictures, and many of them represent body ideals. Physical appearance, in fact, plays an important role on Instagram, and studies have found that adolescents and young people experience distress, are dissatisfied with their bodies, and feel the pressure to look perfect on social media [9], especially when confronted with thin ideals [12,13,14]. Since Instagram provides its users with the opportunity to edit their content before posting it on their profiles, users tend to resort to self-presentation behaviors. In an interview study with 24 teenage girls, Chua and Chang [9] found that, in order to please their followers, social media users tend to match anticipated expectations and preferences by presenting a “highly selective version of themselves” (p. 5). Among female adolescents and young women, self-presentation focuses to a great extent on physical aspects and the idea of beauty. The main reason behind self-presentation is the wish to receive attention for their posts, especially from peers [15]. Yet, posting is not the most prevalent activity on Instagram. In fact, adolescent and young adult Instagram users more often engage in browsing through and “liking” the content on others’ profiles. Longitudinal research found that browsing through other users’ idealized images leads to higher depression levels over time [16]. It can thus be assumed that different forms of engagement on Instagram have differential effects on body dissatisfaction, which is the focus of the present study. More precisely, we wanted to explore which of the following activities on Instagram have a positive relationship with body dissatisfaction (in this study operationalized as a perceived body image discrepancy and a lack of body appreciation): browsing, i.e., looking at other people’s profiles, commenting on other people’s posts, and sharing one’s photos, videos, and stories on the platform.

### 1.2. The Mediating Role of Upward Social Comparison

Browsing, commenting, and posting on Instagram is likely not directly linked to body dissatisfaction, but, as also evidenced in the Facebook Files, through social comparison among teens. According to the Tripartite Influence Model of Body Dissatisfaction and Eating Disturbance [17], young people are influenced by three primary sources: parents, peers, and the media [18]. As a primary socialization agent, parents have a great impact on their daughters’ body image and self-esteem and this can occur, for example, through their comments directly or indirectly related to body weight or appearance in general [19]. As children grow older and enter adolescence, peers become an increasingly important contributor to body image concerns, especially when closeness and conformity are essential components in order to get approval from others [20,21,22]. Adolescents learn from peers what kind of body image is related to popularity and attractiveness [23]. Eventually, the media play a crucial role, because females in the media are often shown with appealing and perfect body proportions [24], which may lead to the development of body image concerns among those who are exposed to them [25]. Nowadays, other than traditional media, social media platforms should be considered. These platforms partly merge socializing agents, as they allow peers to present themselves alongside celebrities and famous influencers.

Past research suggested that particular social media environments such as Instagram may contribute to feelings of inadequacy as a result of *upward* social comparison processes [26,27]. From a social comparison theory perspective, people have a drive to evaluate themselves by comparison with others when objective measures for self-evaluation are lacking [28]. People can do so by engaging in upward and downward comparison. Upward comparison occurs when people compare themselves with someone who is better off [29]. The phrase “better off” may refer to different attributes, including physical appearance. The abundance of selected and manipulated photos, videos, and stories on Instagram provides users with multiple opportunities to engage in upward comparison with others. Research demonstrated that social comparison was directly associated with greater comparison with “ideals” and negative feelings about one’s own body image [30]. In an experimental study, Brown and Tiggemann [10] found that exposure to Instagram images that depict attractive and thin celebrities and peers was associated with higher body dissatisfaction levels, mediated by social comparison. Likewise, Kleemans et al. [31] found that manipulated Instagram pictures had a negative effect on female adolescents’ body image, moderated by social comparison. The number of followers, the number of “likes”, and comments on posted photos or videos provide additional quantitative and qualitative information about the appreciation by others and may thus contribute to the evaluation of the self in comparison with others [32]. In fact, a study revealed that the higher the number of “likes”, the higher the perceived attractiveness of that person, resulting in greater appearance comparison and body dissatisfaction [33]. Eventually, Tiggemann and Anderberg [13] found that comparison with idealized images leads to higher levels of body dissatisfaction, while comparison with images comparing ideals and reality decreased dissatisfaction. Idealized Instagram images are mostly associated with positive emotions, characteristic of the positivity bias on social media [34]. Considering the dominant presence of idealized content on Instagram, the present study focused on the mediating role of upward social comparison in the relationship between different forms of Instagram use (i.e., browsing, commenting, posting) and body dissatisfaction.

#### 1.2.1. Peers as Comparison Targets

Many images and stories on Instagram are posted by peers. A qualitative study by Kenny et al. [23] demonstrated that females tend to imitate those peers who are good-looking in order to feel part of the group. Participants stated that they were more likely to model those peers who received positive comments about their appearance and who got the highest number of “likes” on social media. When referring to peers, it is relevant to distinguish between *close* and *distant* peers. In fact, previous research found that exposure to thin and attractive distant peers had a more detrimental effect than the exposure to close peers, mainly because of the plethora of idealized images of distant peers to which young women were exposed on social media [35]. To follow up on the differential role of these two comparison targets, we further investigated the associations between different types of Instagram use and body dissatisfaction mediated by upward comparison with close and distant peers.

#### 1.2.2. Social Media Influencers as Comparison Targets

Along with peers, Instagram allows users to be directly in contact with people they typically do not know personally, such as social media influencers. Influencers are those people who have been able to establish a strong online presence, which is constantly strengthened through the use of regular posts and their ability to create communities on their social media accounts [36]. One example is Kim Kardashian, one of the most admired female fashion influencers on Instagram [37]. As of December 2021, she had 269 million followers on Instagram. Kardashian’s images consistently present her as “perfect” and very attractive. She has been criticized for this because the high self-esteem expressed by her may be dangerous for young girls who think they are not as good-looking as the influencer [37].

Although social media, and Instagram in particular, are frequently blamed for disseminating idealized body images, voluntarily or involuntarily [38], there is little research to date that examines the relationship between the exposure to female influencers on Instagram and women’s body image. The first study in this regard was carried out by Brown and Tiggemann [10], who demonstrated that the exposure to Instagram images posted by attractive celebrities was related to higher levels of body dissatisfaction among female participants. A similar finding was found in a recent study by Lowe-Calverley and Grieve [11], comparing idealized images of Instagram influencers with high and low popularity metrics (e.g., “likes”, number of followers). The authors found that idealized images led to greater body dissatisfaction, independent of the presence of popularity metrics. However, idealized images are not only accompanied by these metrics, but also by comments representing the appreciation of the viewers. Hu [38] states that comments on idealized images such as “Perfection” or “Body goals” can directly influence women’s self-esteem, and they can be held responsible for the development of expectations and desires among female adolescents and young women that are in contrast to their actual body perceptions, resulting in negative feelings about their own body. Hence, we were interested in whether and how the associations between different types of Instagram use and body dissatisfaction are mediated by upward comparison with social media influencers.

### 1.3. Theoretical Model

**Figure 1:**
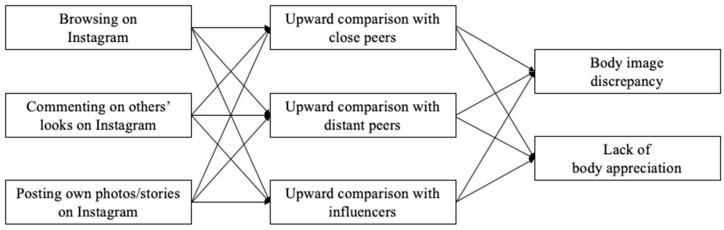
Hypothesized mediation model, all relationships are expected to be positive.

All investigated relationships are summarized in the theoretical mediation model in Figure 1. As depicted in the model, we explored whether different types of Instagram use (i.e., browsing, commenting, posting) are positively related to body dissatisfaction (i.e., body discrepancy, lack of body appreciation) through upward social comparison with close peers, distant peers, and influencers. While we had no a priori assumptions regarding the strength of the relationship between different types of Instagram use and upward social comparison, we expected that the strength of the relationship between upward social comparison and body dissatisfaction would be highest when social media influencers are the comparison target, followed by distant and close peers.

## 2. Materials and Methods

### 2.1. Participants and Procedure

The data for this study were collected through a survey in spring 2019. To be eligible to the study, participants had to be female, less than 30 years of age, and fluent in either Italian or English. Participants were recruited in two different ways: young women aged 18 to 28 were invited to respond to an online survey through posts and snowball sampling on Instagram and Facebook over the course of 30 days. To avoid targeting underage females on social media platforms, female adolescents aged 15 to 17 were asked to fill out a paper-and-pencil survey at a large collaborating high school in Northern Italy. We opted for paper-and-pencil approach since a PC with an Internet connection was not available at school for all adolescent participants and for the dedicated time slot for data collection. The participants were informed beforehand about the aim of the study. Study participation was voluntary, and data were collected in an anonymous form. The Ethics Committee of the university where the research was carried out as well as the headmaster of the collaborating high school approved the study design.

### 2.2. Measures

#### 2.2.1. Instagram Use

Instagram use was assessed using three items. Browsing was assessed with “How often do you look at the looks of others’ in their photos and stories on Instagram?” (1 “never” to 5 “always”), commenting with “How often do you comment on the looks of others in their photos and stories on Instagram?” (1 “never/almost never” to 6 “multiple times a day”), and posting with “How often do you share your photos and stories on Instagram?” (1 “never/almost never” to 6 “multiple times a day”).

#### 2.2.2. Upward Social Comparison

Upward social comparison was measured with three items adapted from Fardouly and Vartanian [35]. The items were translated by the first author, who is an Italian native speaker, and then back-translated by an English native speaker to ensure linguistic equivalence [39]. The introductory question was “When comparing yourself to each of the following people on Instagram, how do you rate yourself?”. A 5-point scale (1 = “Much worse”, 2 = “Worse”, 3 = “Neither worse nor better”, 4 = “Better”, and 5 = “Much better”) was used to assess the extent to which respondents rated themselves as worse or better off compared to a specific target group. The scale was recoded so that higher scores indicated more upward comparison. The different target groups were close friends that participants follow on Instagram and regularly hang out with (close peers), female peers they follow on Instagram but do not regularly hang out with (distant peers), and female social media influencers they follow on Instagram (influencers).

#### 2.2.3. Body Image Discrepancy

The Body Dissatisfaction Scale developed by Mutale et al. [40] was used to assess the discrepancy between the respondents’ actual and ideal body. This scale consists of 9 computer-generated bodies that vary in weight from underweight to overweight. Participants are asked to choose the body they would like to look like (ideal body) and the body they think it is the closest to their perceived actual body shape (actual body). The discrepancy between actual and ideal body represents the participant’s body image discrepancy score. Negative values indicate that respondents consider their actual body thinner than their ideal body, a value of 0 indicates no discrepancy, and positive values indicate that the actual body is perceived bigger than the ideal body.

#### 2.2.4. Body Appreciation

The Body Appreciation Scale [41] was used to measure body dissatisfaction beyond a perceived body image discrepancy. Again, forward-backward translation was done to ensure linguistic equivalence. The scale comprises 13 items (e.g., “I feel good about my body.”). Participants were asked to select a response option for each of the items, choosing on a 5-point Likert scale (1 = “Never”, 2 = “Seldom”, 3 = “Sometimes”, 4 = “Often”, or 5 = “Always”). All items were reverse coded and averaged to obtain an overall score for the lack of body appreciation, i.e., the higher the score, the lower the participant’s body appreciation. Reverse coding facilitated the interpretation of this concept alongside body image discrepancy, where both can be considered indicators of body dissatisfaction.

#### 2.2.5. Covariates

Covariates included participants’ age, which was collapsed into 1 = “adolescents” and 0 = “young adults”. This variable simultaneously captured the two different assessment modes (i.e., paper-and pencil survey for adolescents and online survey for young adults). Furthermore, the analysis considered participants’ body mass index (BMI), calculated from self-reported height and weight. For participants up to 19 years of age, we calculated age- and gender-specific z-scores using the World Health Organization (WHO) growth reference [42]. For older participants, we calculated z-scores using the formula BMI = weight (in kg)/height (in meters)^2.

### 2.3. Analytical Plan

SPSS was used to conduct the preliminary statistical analyses and impute missing data. Missing data were imputed using an expectation-maximization algorithm on a dataset including only participants with less than two missing data points on the variables included in the final mediation models. Descriptive statistics were calculated for all measures to assess normal distribution and detect outliers. Skewness and kurtosis values in the absolute range of two were considered acceptable. Scale reliability was assessed with Cronbach’s alpha. Bivariate correlations among all the variables were conducted. The lavaan package [43] in R [44] was used to conduct confirmatory factor analysis on the two final outcome measures of interest (i.e., body image discrepancy and body appreciation) and to test the hypothesized mediation model in Figure 1. A robust maximum likelihood (MLR) estimator was used. A model was deemed good-fitting if at least two of the following thresholds were met: CIF ≥ 0.95, RMSEA ≤ 0.06, and SRMR ≤ 0.08 [45].

## 3. Results

### 3.1. Preliminary Results

Survey data were obtained from 354 females. The analytical sample only included females with an Instagram account and two or fewer missing data points on the variables included in the final model (*n* = 291; 82.2%). The average age of the final sample was 19.8 (*SD* = 4.57). Given the bimodal distribution of age, the variable was collapsed into adolescents (*n* = 136; 46.7%) and young adults (*n* = 155; 53.3%). The dichotomous age variable further reflected the two different data collection procedures (adolescent sample = paper-pencil survey; young adult sample = online survey). The large majority of participants were Italian (*n* = 276; 94.8%). The interquartile range of age-specific z-scores for BMI was 1.19. According to the growth standards of the WHO [42], 1% (*n* = 3) of females in the final sample were underweight, 83% (*n* = 242) were normal weight, 13% (*n* = 38) were overweight, and 3% (*n* = 8) were obese. Concerning weekly Instagram use, the majority declared spending more than five hours per week using the platform (*n* = 118; 40.5%), followed by three to five hours (*n* = 74; 25.4%) and one to two hours (*n* = 43; 14.8%). Regarding the different types of Instagram use, more than half of the participants reported often or always browsing through others’ looks in photos or stories (*n* = 160; 54.9%), while only a minority commented on others’ looks on a daily basis (*n* = 49; 16.8%). Approximately one in four females posted her own photos or stories on Instagram (*n* = 76; 26.2%).

An initial confirmatory factor analysis on the two final outcome measures of interest (i.e., body image discrepancy and lack of body appreciation) revealed that the two items “I am attentive to my body’s needs” and “I engage in healthy behaviors to take care of my body” as part of the (lack of) Body Appreciation Scale were indicators of a distinct concept. They were thus eliminated, which resulted in good model fit (χ^2^ (54) = 121.762, *p* ≤ 0.001; CFI = 0.963; RMSEA = 0.066; SRMR = 0.044). The final measure for the lack of body appreciation thus included eleven items with factor loadings ranging from 0.282 to 0.891 (see Table 1). Given the good overall model fit of the final model, we decided to keep the item with the lowest loading (i.e., “I do not focus a lot of energy being concerned with my body shape or weight”). Cronbach’s alpha was α = 0.91. The correlation coefficient between the two latent concepts (i.e., body image discrepancy and lack of body appreciation) describing body dissatisfaction was *r* = 0.388, *p* < 0.001.

Bivariate correlations (Table 2) did not reveal any significant relationships between different types of Instagram use (i.e., browsing, commenting, posting) and body image discrepancy or lack of body appreciation. However, browsing on Instagram and commenting on others’ looks were significantly positively related to upward comparison with social media influencers. Upward social comparison was significantly positively associated among the three different comparison targets, the reverse-coded body appreciation scale, and to a lesser extent body image discrepancy, except for the relationship between upward comparison with distant peers and body image discrepancy, which was not significant. Given that being an adolescent and BMI z-scores showed significant relationships with body discrepancy and lack of body appreciation, both were added as covariates on the final outcome variables in the mediation model.

### 3.2. Final Model Results

**Figure 2:**
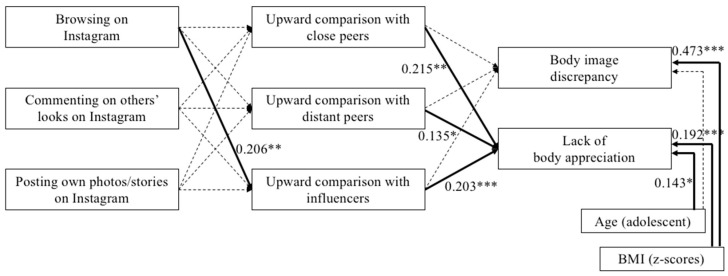
Final mediation model, * *p* < 0.05; ** *p* < 0.01; *** *p* < 0.001. Only significant standardized path coefficients for continuous variables are displayed. Dotted lines display non-significant paths.

The tested mediation model showed good model fit (χ^2^ (12) = 23.374, *p* = 0.025; CFI = 0.963; RMSEA = 0.057; SRMR = 0.042). Among the three different types of Instagram use (i.e., browsing, commenting, and posting), browsing through other people’s looks on Instagram turned out to be significantly and positively associated with upward comparison when social media influencers were the comparison target. The model did not include significant associations between browsing on Instagram and upward comparison with close or distant peers, and neither did it include significant associations between commenting or posting on Instagram and upward comparison with close peers, distant peers or influencers. In turn, controlling for being an adolescent and BMI (measured with age-specific z-scores), upward comparison with the three different comparison targets was significantly and positively associated with a lack of body appreciation, while it was not related to perceived body discrepancy. Being a female adolescent (compared to a young woman) and higher BMI were related to worse body appreciation. Furthermore, higher BMI, but not being an adolescent, was associated with higher levels of body image discrepancy. Eventually, the indirect association between browsing on Instagram and lack of body appreciation, through upward comparison with influencers, was positive and significant (B = 0.029, SE = 0.012, β = 0.042, *p* = 0.011). The coefficients of all direct paths and correlations among endogenous variables are summarized in Table 3. The final model (Figure 2) explains 23% of the variance in the lack of body appreciation and perceived body image discrepancy.

## 4. Discussion

Instagram is a highly popular social media platform among female adolescents and young women. It puts an emphasis on visual representations in the form of pictures, videos, and stories, which can be “polished” thanks to the use of in-built filters and other editing applications. The platform’s characteristics led researchers and the public alike to voice concerns about a possible detrimental effect of Instagram use on females’ body image and subsequent unhealthy dieting and exercising behaviors. To shed light on the relationship between Instagram use and body dissatisfaction, the present study used theoretical insights from the Tripartite Influence Model of Body Dissatisfaction and Eating Disturbance [17] as well as Social Comparison Theory [28] and tested a mediation model linking different types of Instagram use (i.e., browsing through others’ profiles, commenting on others’ looks, and posting one’s own photos and stories) to body dissatisfaction (i.e., body image discrepancy and lack of body appreciation), mediated by upward social comparison with close peers, distant peers, and social media influencers.

We found evidence of a link between browsing through the looks of others and lower levels of body appreciation, fully mediated by upward social comparison with social media influencers. Interestingly, body appreciation, not perceived body discrepancy, was linked to upward social comparison, showing that body appreciation and perceived body discrepancy are distinct indicators of body dissatisfaction. We can thus conclude that browsing through social influencers’ Instagram profiles as a form of orientation towards beauty ideals is associated with detrimental outcomes, as previously found in the context of social media use and depression in adolescents [27], while commenting or posting one’s own photos or stories is not. The latter two are less frequent activities among the participants in our sample, and it remains subject to future studies to explore whether and how these more active types of Instagram use are associated with body dissatisfaction. This requires an even more nuanced look at which types of comments (e.g., appreciation or criticism of the look of influencers) and which types of photos or stories being shared (e.g., edited vs. not) are associated with body dissatisfaction. For example, it has been argued that young females seek attention by sharing edited photos of themselves where they look nicer to increase their self-esteem and control their level of insecurity [15], and this can be the consequence of higher body dissatisfaction (i.e., posting edited photos as a coping strategy), as well as the cause of such posting practices. To unravel the directionality, future research should apply random-intercept cross-lagged panel models [46,47] that allow separating within-person effects (i.e., the causal mechanisms of interest) from between-person effects (i.e., associations across study participants).

The mediating role of social comparison with social media influencers supports findings from past experimental studies showing that influencers on Instagram have a negative impact on women’s body image and that social comparison processes need to be considered [10,11,13]. When it comes to peers as the comparison target, a different picture emerged: while upward social comparison with close and distant peers was significantly and positively related to lower levels of body appreciation, the comparison with these two targets was not related to any type of Instagram use investigated in the present study. This finding underlines that peers are indeed comparison targets, but more so in offline or other online contexts, and this stands in contrast to a prior study which found that exposure to idealized images of distant peers on Instagram is related to social comparison and worse mood and body image [10]. Thus, social media influencers, not peers, are an increasingly important socializing agent, which contribute to feelings of inadequacy as a result of upward social comparisons [26,27].

With regard to the covariates studied in our mediation model, it should be noted that younger females aged 15 to 17 reported significantly lower levels of body appreciation than young women. This echoes findings from past studies demonstrating that girls in late adolescence report higher levels of body dissatisfaction [48]. As females go through puberty, their body changes considerably. This physical change goes hand in hand with a greater attention to and need for peer approval. Indeed, those whose self-worth is contingent on others’ approval experience lower levels of body esteem [49], which is yet another indicator of body dissatisfaction. As females grow older, they become less likely to attach physical appearance to their self-worth [50]. Eventually, we found that females with a higher BMI reported higher levels of body image discrepancy as well as a greater lack of body appreciation, indicating, once again, that today’s beauty ideal is, to a large extent, still determined by thinness.

### Limitations and Future Directions

The present study comes with some limitations. First, a cross-sectional design was used, which does not allow for drawing conclusions on the causal mechanisms between the investigated variables. A longitudinal or experimental design would overcome this limitation. Yet, our hypothesized mediation model was based on prior research on the topic, both cross-sectional and longitudinal or experimental. Furthermore, we tested an alternative mediation model reversing the hypothesized paths between Instagram use and body dissatisfaction, which led to a significant deterioration of all fit indices. Second, our sampling method limits the generalizability of the findings to a larger female population and the final sample size was comparably small. A follow-up power analysis with the semPower package [51] revealed that, for a structural equation model with a target RMSEA of at least 0.06, an alpha of 0.05, 12 degrees of freedom—resulting from our final model with age and BMI as covariates—and a desired power of 0.80, we would have needed a final sample size of *n* = 399 to detect small yet significant associations. For this reason, future studies should include bigger samples and apply rigorous probability sampling techniques. Third, all measures are based on self-report data, which are subject to different biases. Both estimation bias and social desirability bias may have systematically influenced the data. In this regard, it would be interesting to combine content-analyzed data from Instagram accounts with objectively tracked usage data and self-report perceptions of appearance anxiety and body dissatisfaction. However, privacy and legal restrictions challenge such a methodological approach in European countries. Fourth, some concepts in the final mediation models were measured with single-item indicators (e.g., Instagram use, upward social comparison), though multi-item indicators should be preferred because of their greater stability. Fifth and last, we did not consider additional psychological characteristics such as self-esteem or negative affect [52,53] as moderators, which are likely to amplify the associations between the concepts included in our study for vulnerable females. To inform targeted public health interventions, future studies should include personal characteristics as additional moderators to provide a more nuanced understanding of the conditions under which the mediated associations between Instagram use and dissatisfaction hold or are more pronounced.

## 5. Conclusions

In conclusion, the present study found that the relationship between browsing through the looks of others on Instagram and body dissatisfaction, measured by the lack of body appreciation, is fully mediated by upward appearance comparison with social media influencers. Thus, the exposure to idealized pictures and stories of this comparison target is associated with detrimental outcomes in female adolescents and young women. The findings of our study highlight the need for public health interventions to raise awareness about the posting practices of social media influencers and to strengthen a positive body image, with special attention to particularly vulnerable girls. Prior interventions to promote a positive body image among women proved to be effective [54]. However, interventions aimed at female adolescents and young women, such as the *Boost Body Confidence and Social Media Savvy* intervention [55], should specifically consider the social media context and highlight the nature and detrimental consequences of the exposure to manipulated photos and stories. Interventions supported by social media influencers can be particularly useful. In fact, body positivity movements already initiated by some celebrities on social media [56] can help females to focus less on the external beauty ideals conveyed through social media and to foster self-esteem and create emotional support, thus preventing and addressing health issues such as body image concerns. In addition to that, media literacy interventions providing factual information and debunking false beliefs [57] as well as strengthening the ability to access, analyze, and evaluate body-image-related content [58], can be another successful strategy to counteract unrealistic images of female beauty and to help females to think critically about the idealized body images and messages they find on social media.

## Figures and Tables

**Table 1:** CFA results for items of the final Body Appreciation Scale.

Items	M	SD	Factor Loading	SE	CILO	CIHI
On the whole, I am satisfied with my body.	2.99	1.04	0.891	0.015	0.861	0.921
My feelings toward my body are positive, for the most part.	2.95	1.03	0.884	0.015	0.854	0.914
Despite its imperfections, I still like my body.	2.87	1.05	0.871	0.018	0.837	0.906
I feel good about my body.	2.92	1.01	0.869	0.018	0.835	0.904
I take a positive attitude toward my body.	2.73	1.03	0.843	0.020	0.803	0.883
Despite its flaws, I accept my body for what it is.	2.82	1.09	0.813	0.024	0.766	0.861
I feel that my body has at least some good qualities.	2.38	1.00	0.727	0.029	0.670	0.784
I respect my body.	2.25	0.90	0.609	0.041	0.528	0.690
My self-worth is independent of my body shape or weight.	3.18	1.15	0.439	0.054	0.333	0.545
I do not allow unrealistically thin images of women presented in the media to affect my attitudes toward my body.	2.93	1.24	0.429	0.053	0.324	0.533
I do not focus a lot of energy being concerned with my body shape or weight.	3.31	1.10	0.282	0.064	0.157	0.408

Note: All items are reverse coded with a final scale range from 1 “Always” to 5 “Never”, CILO = lowest level CI, CIHI = highest level CI.

**Table 2:** Descriptive statistics and bivariate Pearson’s correlations (*n* = 291).

Variables	M (SD)	1	2	3	4	5	6	7	8	9	10
1. Age (adolescent)	0.47 (0.50)	-									
2. BMI z-scores	0.02 (0.98)	−0.15 **	-								
3. Body image discrepancy	1.11 (1.17)	0.02	0.47 **	-							
4. Lack of body appreciation	2. 85 (0.77)	0.15 *	0.23 **	0.37 **	-						
5. Upward comparison with close peers	3.09 (0.59)	0.12 *	0.13 *	0.13 *	0.38 **	-					
6. Upward comparison with distant peers	3.18 (0.76)	0.05	0.11	0.09	0.34 **	0.46 **	-				
7. Upward comparison with influencers	3.62 (0.89)	0.02	0.07	0.13 *	0.33 **	0.32 **	0.39 **	-			
8. Browsing on Instagram	3.49 (1.08)	0.01	−0.18 **	0.09	0.10	−0.05	−0.06	0.24 **	-		
9. Commenting on others’ photos/stories on Instagram	2.14 (1.78)	0.20 **	−0.01	0.02	0.07	0.06	0.05	0.14 *	0.31 **	-	
10. Posting own photos/stories on Instagram	3.78 (1.30)	−0.20 **	0.03	0.04	0.02	−0.03	0.00	0.10	0.27 **	0.24 **	-

Note: ** *p* < 0.01 (2-tailed); * *p* < 0.05 (2-tailed).

**Table 3:** Path and correlation coefficients for all direct associations in the final mediation model.

Direct Associations	B	SE	β	*p*
Browsing TO Upward comparison with close peers	−0.037	0.028	−0.067	0.181
Commenting TO Upward comparison with close peers	0.028	0.026	0.084	0.271
Posting TO Upward comparison with close peers	−0.013	0.034	−0.029	0.696
Browsing TO Upward comparison with distant peers	−0.058	0.039	−0.082	0.137
Commenting TO Upward comparison with distant peers	0.032	0.031	0.076	0.291
Posting TO Upward comparison with distant peers	0.001	0.035	0.002	0.976
Browsing TO Upward comparison with influencers	0.170	0.050	0.206	0.001
Commenting TO Upward comparison with influencers	0.036	0.030	0.071	0.242
Posting TO Upward comparison with influencers	0.019	0.042	0.028	0.646
Upward comparison with close peers TO Body image discrepancy	0.077	0.120	0.039	0.521
Upward comparison with distant peers TO Body image discrepancy	−0.029	0.102	−0.019	0.779
Upward comparison with influencers TO Body image discrepancy	0.124	0.064	0.095	0.051
Age (adolescent) TO Body image discrepancy	0.193	0.123	0.83	0.118
BMI (z-scores) TO Body image discrepancy	0.564	0.072	0.473	<0.001
Upward comparison with close peers TO Lack of body appreciation	0.273	0.090	0.215	0.002
Upward comparison with distant peers TO Lack of body appreciation	0.135	0.057	0.135	0.018
Upward comparison with influencers TO Lack of body appreciation	0.173	0.045	0.203	<0.001
Age (adolescent) TO Lack of body appreciation	0.216	0.084	0.143	0.010
BMI (z-scores) TO Lack of body appreciation	0.149	0.038	0.192	<0.001
Upward comparison with close peers WITH Upward comparison with distant peers	0.203	0.037	0.458	<0.001
Upward comparison with close peers WITH Upward comparison with influencers	0.170	0.036	0.335	<0.001
Upward comparison with distant peers WITH Upward comparison with influencers	0.269	0.041	0.416	<0.001
Body image discrepancy WITH Lack of body appreciation	0.190	0.042	0.281	<0.001

Note: χ^2^ (12) = 23.374, *p* = 0.025; CFI = 0.963; RMSEA = 0.057 (90 CILO% = 0.021, 90 CIHI% = 0.094); SRMR = 0.042.

## Data Availability

The dataset is available at https://osf.io/kd957/ (accessed on 20 December 2021).
